# Evaluation of the Safety of Recombinant Zoster Vaccine on Patients with Systemic Lupus Erythematosus in a Tertiary Hospital in Saudi Arabia: A Retrospective Single Centre Study

**DOI:** 10.31138/mjr.140126.sqy

**Published:** 2026-06-12

**Authors:** Hoda Draz, Nada Ibrahim, Ibrahim Al-Homood, Assia S. Alwabel

**Affiliations:** 1 Internal Medicine Department, Rheumatology Section, KFMC, Riyadh, Alfaisal University, Faculty of Medicine, Riyadh, Saudi Arabia;; 2 Rheumatology Department, Benha Faculty of Medicine and University Hospitals, Egypt

**Keywords:** systemic lupus erythematosus, Recombinant Zoster Vaccine, Biological therapy, immunocompromise

## Abstract

**Introduction:**

Herpes Zoster (HZ) infection is more severe in patients with Systemic lupus erythematosus (SLE). Recent guidelines recommend the Recombinant Zoster Vaccine (RZV) for prevention of HZ in immunocompromised adults aged ≥18 years. RZV has demonstrated efficacy against HZ infection in immunocompromised populations, but data regarding its safety among SLE patients are lacking, especially those who are on biological therapy.

**Objective:**

Evaluation of the safety of RZV among SLE patients, including patients on biological therapy.

**Method:**

A retrospective study of 170 patients with SLE who received at least one dose of RZV and 45 unvaccinated SLE patients were included. Data were extracted from the King Fahad Medical City (KFMC) hospital database system. Data analysis was carried out using Statistical methods.

**Results:**

A study involved 170 SLE patients who received RZV (90.6% females, with a mean age of 38.5 years and an SLE duration of 8.5±6.2 years). Seventy percent completed two doses of RZV. The mean baseline, post-first-dose, and post-second-dose SLEDAI scores were 3.43±5.27, 3.31±3.11, and 2.97±2.78, respectively, indicating no significant change. A subset of patients was treated with biological therapies, including Belimumab, Rituximab, and Anifrolumab. The incidence of SLE flare within 90 days was 12.9% after the first dose and 12.3% after the second dose. There was a statistically significant association between higher baseline SLEDAI scores and flares after both doses.

**Conclusion:**

Vaccination with RZV in patients with SLE is safe and not associated with increased SLE activity which is comparable with unvaccinated patients. The reported side effects were mild and comparable to those of the general population.

## INTRODUCTION

Systemic lupus erythematosus (SLE) is a chronic autoimmune disease with variable multisystem manifestations, commonly occurring among adolescent females and young women.^1^ SLE patients are considered immunocompromised due to both disease-related immune dysfunction and immunosuppressive therapies; thus, they are at a greater risk for herpes zoster infection (HZ).^2^ HZ, also known as shingles, is a viral infection secondary to reactivation of Varicella-Zoster Virus, which establishes latency in sensory ganglia.^3^ Multiple studies showed that SLE patients have higher incidence rates of HZ infection, often with more severe disease and a high risk of complications. One study in Japan showed a 1.8–8.4-fold higher risk of HZ infection in SLE patients compared to other patients. Another meta-analysis showed that HZ infection was the commonest opportunistic infection, with an incidence of 22 cases per 1000 per year.^5^ Several risk factors contributed to the increased risk of HZ in SLE patients, including the use of high doses of glucocorticoids, biological therapy, lymphopenia, and renal involvement, especially lupus nephritis, which were the most reported risk factors.^4,5^ Higher rates of complications were observed in SLE patients post HZ infection, such as post-herpetic neuralgia which was reported in 15.2% compared to 5–9% in the general population. Also, SLE patients have experienced high risk of superimposed bacterial infections in about 8.7 % in comparison to 1.4 in that of general population.^5^ Herpes Zoster Vaccine (HZV) was developed to reduce disease-related health burden and its complications. Currently, two forms of the vaccine are available: live attenuated and recombinant adjuvanted. The recombinant vaccine is recommended for immunocompromised patients by the Advisory Committee on Immunisation Practices (ACIP) and the American College of Rheumatology (ACR).^2,3^ It is an adjuvanted subunit administered in two doses, which demonstrated a high efficacy in preventing herpes zoster infection in both healthy adults and immunocompromised patients.^6^ This vaccine is considered safe, with most reported side effects being local injection site reactions, such as pain, swelling, and redness. In addition to systemic manifestations, including fatigue, myalgia, and headache.^7^ A study was done in the United States of America (USA) about the effectiveness and safety of the RZV in adult patients with SLE, in which they found that the vaccine is not associated with severe SLE flare-ups.^8^ Due to paucity of data regarding RZV safety and its side effects, especially on SLE patients, further research is recommended. This study aims to evaluate the vaccine’s efficacy and short-term side effects among SLE patients in Saudi Arabia.

## METHODS

### Study design, area, population, and sampling

This is a retrospective study done at a tertiary hospital in Riyadh, KFMC, Saudi Arabia.

Inclusion criteria:

- We included all the patients who are ≥18 years and received RZV, with confirmed SLE diagnosis according to the classification criteria established by the Systemic Lupus International Classification Committee (SLICC)*.^11^*
- We also included 45 confirmed SLE patients who did not receive the vaccine as control group

Exclusion criteria:

- Patients with active disease (SLEDAI ≥12) flare at the time of vaccination, pregnant SLE patients, and patients on high doses of corticosteroid (≥20 mg per day).
- Disease activity was assessed using the Systemic Lupus Erythematosus Disease Activity Index 2000 (SLEDAI-2K), with scores 1–5 considered as mild, 6–11 as moderate, and 12 or more as severe.^12^

Data were collected from the hospital database system’s medical records that met our inclusion criteria from July 2024 to July 2025 including Comprehensive background checks, laboratory investigations, and immunological tests.

### Ethical considerations

The study protocol was approved by the local ethics committee in KFMC. Written informed consent was obtained from all patients prior to vaccination.

### Statistical analysis

SPSS version 28 was used for data administration and statistical analysis (IBM, Armonk, NY, USA). Descriptive statistics were performed, and categorical variables were frequency distributions and percentages. The chi-square test or Fisher’s exact test was used to compare the categorical data. Numbers were reported as means and standard deviations or medians and ranges in accordance with the assumption of normality. Test of Kolmogorov- Smirnov and Shapiro Wilk were used to test normality of distribution of numerical variables. Mann Whitney test (U) to test significance difference of non-parametric quantitative variables between two groups. Friedman’s test was used when comparing repeated measures taken of more than two readings of not normally distributed data, then Wilcoxon signed-rank test was used for pair-wise comparisons. All statistical analysis was unidirectional. Significant results were determined at *P* ≤ 0.05 and highly significant at *P* ≤ 0.001.

## RESULTS

The study population is overwhelmingly female, accounting for 82.2–90.6% of the participants. This is typical of systemic lupus erythematosus (SLE) studies, as the disease predominantly affects women. The mean age of the patients is 38.55–39.27 years.

The mean disease duration is 8.54 years. About one-third of the patients (35.9%, 33.3%) have a history of Lupus Nephritis.

Nineteen patients (11.2%) had a history of HZ infection. This is the population at risk that the RZV vaccine targets. For those with a prior infection, the mean time between zoster infection and vaccination was 3.57 years. There is no statistically significant difference between both groups regarding the demographic data and other parameters (**Table 1**).

**Table 1.** Demographic data of patients with SLE who received the RZV, disease profile, previous HZ infection history and controls.

**Variable**	**Cases (n=170)**	**Control (n=45)**	**Test of significance**	**P value**

**Gender**				
Male	16 (9.4%)	8 (17.8%)		0.113
Female	154 (90.6%)	37 (82.2%)	X2= 2.511	

**Age (years)**				
Mean ± SD	38.55±11.57	39.27±12.858	U= 3639.00	0.791
median (Min-Max)	37.0 (18–68)	37.0 (18–63)		

**Disease duration (years)**				
Mean ± SD	8.54±6.2	8.66±7.91	U=3373.50	0.737
median (Min-Max)	7 (1–47)	7 (1–47)		

**Lupus Nephritis**				
Yes	61 (35.9%)	15 (33.3%)	X2=0.101	0.750
No	109 (64.1%)	30 (66.6%)		

**History of zoster infection**				
Yes	19 (11.2%)	0	FEX=5.571	0.016*
No	151 (88.8%)	45(100%)		

SD: Standard deviation, Min: Minimum- Max: Maximum, X2: Chi square test, U: Mann Whitney test, FEX: Fisher Exact test,

*: significant at p<0.05.

Among vaccination side effects, local pain at the injection site was the most common, reported by 15.3% of patients. Fatigue is the most common systemic side effect, reported by 14.7% of patients, 12.4% of patients reported fever, 11.2% of patients reported myalgia (muscle pain). Local swelling and redness were reported in 7.6% of cases. Only 1.2% (n=2) of the patients developed a Zoster infection after vaccination, suggesting a very high level of short-term vaccine effectiveness in this cohort (**Table 3**).

**Table 3.** Vaccination side effect.

**Side Effects**	**N (%) (n=170)**

**Fever**	
Yes	21 (12.4%)
No	149 (87.6%)

**Fatigue**	
Yes	25 (14.7%)
No	145 (85.3%)

**Myalgia**	
Yes	19 (11.2%)
No	151 (88.8%)

**Local pain**	
Yes	26 (15.3%)
No	144 (84.7%)

**Local swelling erythema**	
Yes	13 (7.6%)
No	157 (92.4%)

**Zoster infection after vaccination**	
Yes	2 (1.2%)
No	168 (98.8%)

The mean baseline SLEDAI score is (3.43±2.27), with the median baseline 2.98. The mean SLEDAI score after the first dose (3.31±3.11) is almost similar to the mean baseline SLEDAI. One-hundred twenty-two patients (71.76%) completed the two-dose series. The mean SLEDAI score after the second dose (2.97±2.78) is also consistent with the baseline and post-first-dose mean scores. The median SLEDAI (3) remains low after the second dose.

The test of significance, Friedman’s test, shows no statistically significant difference in the mean SLEDAI scores across the three time points (Baseline, Post-Dose 1, Post-Dose 2), with a P-value of 0.133. This suggests that RZV vaccination did not cause a sustained change in the mean SLEDAI score. No significant difference between Baseline, Post-1^st^, and 2^nd^ doses SLEDAI (P-value > 0.05).

In the control group, unvaccinated SLE patients have comparable results of SLEDAI scores with no statistically significant difference between both groups, while there is significant reduction in repeated measures of SLEDAI score in control group only with P values of 0.035, 0.026 and 0.021 (**Table 4**).

**Table 4.** Comparing mean SLEDAI scores at baseline, after 1st dose (3 months duration), and after 2nd dose (6 months duration) to identify any sustained changes in disease activity.

**Side Effects**	**N (%) (n=170)**

**Fever**	
Yes	21 (12.4%)
No	149 (87.6%)

**Fatigue**	
Yes	25 (14.7%)
No	145 (85.3%)

**Myalgia**	
Yes	19 (11.2%)
No	151 (88.8%)

**Local pain**	
Yes	26 (15.3%)
No	144 (84.7%)

**Local swelling erythema**	
Yes	13 (7.6%)
No	157 (92.4%)

**Zoster infection after vaccination**	
Yes	2 (1.2%)
No	168 (98.8%)

SD: Standard deviation, Min: Minimum- Max: Maximum, U: Mann Whitney test, X2 (F): Friedman test, Z: Wilcoxon Signed Ranks Test,

*: significant at p<0.05.

The highly significant P-value confirms that higher Baseline SLEDAI and SLEDAI 2 after the first dose are strong and significant risk factors for flare after the first and second doses. The association between Steroid use and flare after the first and second doses is statistically significant (P=0.023) and (P=0.016), respectively. A higher proportion of patients who flared (50–60 %) were on steroids compared to those who did not flare (26.4–27.1%). This suggests that steroid use is a risk factor for flare after both first and second doses in this univariate analysis (**Table 5**).

**Table 2.** Medication history of the patients of both groups.

**Immunosuppressant medication**	**Cases (n=170)**	**Control (n=45)**	**Test of significance**	**P value**

**Hydroxychloroquine**				
Yes	147 (86.5%)	42 (93.3%)	X2=1.576	0.209
No	23 (13.5%)	3 (6.7%)		

**Azathioprine**				
Yes	45 (26.5%)	10 (22.2%)	X2=0.337	0.561
No	125 (73.5%)	35 (77.8%)		

**Methotrexate**				
Yes	20 (11.8%)	3 (6.7%)	FEX= 0.968	0.423
No	150 (88.2%)	42 (93.3%)		

**Mycophenolate Mofetil**				
Yes	40 (23.5%)	16 (35.6%)	X2= 2.672	0.102
No	130 (76.5%)	29 (64.4%)		

**Belimumab**				
Yes	24 (14.1%)	5 (11.1%)	X2=0.276	0.600
No	146 (85.9%)	40 (88.9%)		

**Rituximab**				
Yes	6 (3.5%)	0 (0%)	FEX= 1.634	0.348
No	164 (96.5%)	45 (100%)		

**Anifrolumab**				
Yes	3 (1.8%)	0 (0%)	FEX= 0.805	1.000
No	167 (98.2%)	45 (100%)		

**Calcineurin inhibitors (Tacrolimus)**				
Yes	3 (1.8%)	0 (0%)	FEX= 0.805	1.000
No	167 (98.2%)	45 (100%)		

**Steroid**				
Yes	51 (30.0%)	20 (44.4%)	X2= 3.356	0.067
No	119 (70.0%)	25 (55.6%)		

**Steroid Dose (n=51)**				
2.5 mg	1 (2%)	5 (25%)		
5 mg	42 (84%)	10 (50%)		
7.5 mg	1 (2%)	2 (10%)	FEX= 20.195	0.001**
10 mg	5 (10%)	0 (0%)		
15 mg	1 (2%)	2 (10%)		
35 mg	0 (0%)	1 (5%)		

**Steroid Dose (n=51)**				
Mean ± SD	5.69±2.07	7.13±7.45	U=468.50	0.487
median (Min-Max)	5 (2.5–15)	5 (2.5–35)		

SD: Standard deviation, Min: Minimum- Max: Maximum, X2: Chi square test, U: Mann Whitney test, FEX: Fisher Exact test,

*: significant at p<0.05.

**Table 5.** Analysing the association of studied factors with post-vaccination flare risk after the first and second doses in cases (n=170).

	**Flare risk after first dose**		**Test of significance**	**P Value**	**Flare risk after second dose**		**Test of significance**	**P Value**
	
**Variable**	**Yes (n=22)**	**No (n=148)**			**Yes (n=15)**	**No (n=107)**		

**Baseline SLEDAI**								
Mean ± SD median (Min-Max)	7.36±1.80 8.0 (4–10)	4.33±9.79 2.0 (0–12)	**U= 174.00**	<0.001**	4.93±2.465.0 (0–9)	3.69±6.373.50(0–12)	**U=492.50**	0.016*

**SLEDAI 2 (after 1st dose)** Mean ± SD median (Min-Max)					6.47±2.85 7.0 (2–12)	3.04±3.04 2.0 (0–14)	**U=301.50**	<0.001**

**Gender** (Female)	18 (81.8%)	135 (91.2%)	FEX= 2.404	0.335	13 (86.7%)	98 (91.6%)	FEX= 1.603	0.437

**Age** (years)	35 (19–65) 35.5±10.91	35.5 (22–54) 36.2±9.37	U=439.50	0.376	36 (19–56) 35.75±9.6	(22–44) 34.5±9.6538	U=4399.50	0.376

**Disease duration** (years)	8.2±4.73 6 (3–17)	8.18±9.36 5.0 (2–47)	U=506.50	0.881	7.0±4.72 6 (2–15)	8.96±9.56 5.5 (1–47)	U=506.5	0.881

**Lupus Nephritis** (Yes)	9 (40.9%)	52 (35.4 %)	X2= 0.254	0.614	9 (60%)	40 (37.7%)	X2= 2.703	0.100

**History of zoster infection before** (Yes)	0 (0%)	19 (12.8 %)	FEX=3.777	0.255	1 (6.7%)	13 (12.1%)	FEX=0.841	1.000

**Plaquenil** (Yes)	18 (81.8%)	128 (86.5%)	FEX= 0.344	0.521	14 (93.3%)	90 (84.1%)	FEX=0.88	0.696

**Imuran** (Yes)	9 (40.9%)	35 (23.6%)	X2=2.974	0.085	5 (33.3%)	27 (25.2%)	FEX=0.44	0.536

**MTX** (Yes)	3 (13.6%)	17 (11.5%)	FEX=0.085	0.727	1 (6.7%)	11 (10.3%)	FEX=0.19	1.000

**MMF** (Yes)	6 (27.3%)	34 (23.0%)	X2=0.197	0.657	6 (40%)	21 (19.6%)	FEX=3.16	0.097

**Belimumab** (Yes)	4 (18.2%)	20 (13.5%)	FEX=0.344	0.557	3 (20%)	14 (13.1%)	FEX=0.52	0.438

**Rituximab** (Yes)	0 (0%)	6 (4.1%)	FEX=0.925	1.000	0 (0%)	2 (1.9%)	FEX=0.28	1.000

**Anifrolumab** (Yes)	0 (0%)	3 (2.0%)	FEX= 0.454	1.000	0 (0%)	2 (1.9%)	FEX=0.32	1.000

**CNI (tacrolimus)** (Yes)	1 (4.5%)	2 (1.4%)	FEX=1.113	0.344	1 (6.7%)	1 (0.9%)	FEX=2.64	0.233

**Steroids** (Yes)	11 (50%)	39 (26.4%)	**X2= 5.159**	0.023*	9 (60%)	29 (27.1%)	**FEX=6.63**	0.016*

**Steroid dose**	4.75±0.79 5 (3–5)	5.96±2.3 5 (5–15)	U=171	0.091	5.94±3.77(3–15)	5.63±1.69(5–10)	U=129.0	0.857

SD: Standard deviation, Min: Minimum- Max: Maximum, X2: Chi square test, U: Mann Whitney test, FEX: Fisher Exact test,

*: significant at p<0.05,

**: highly significant at p<0.01.

Logistic Regression model confirmed previous results that SLEDAI baseline and steroid use were significant predictors of flare after the first dose of vaccination, but in a multivariate model, only SLEDAI baseline remained a significant predictor, p=0.005** (**Table 6**).

**Table 6.** Regression model for analyzing the probable risk factor of flare after the first dose in Cases (n=170).

	**Univariate logistic regression model**			**Multivariate logistic regression model**						
	**OR**	**95% (C.I)**	**Significance**	**OR**	**95% (C.I)**	**Significance**
**Baseline SLEDAI score**	1.293	1.100–1.521	0.002**	1.266	1.074–1.492	0.005*
**Steroids**	2.795	1.122–6.959	0.027*	1.975	0.744–5.238	0.172

OR: Odds Ratio, C.I: Confidence Interval,

*: significant at p<0.05,

**: highly significant at p<0.01.

## DISCUSSION

Although there is a well-established data about the safety and effectiveness of RZV, yet there is not enough evidence about its safety among patients with SLE. Patients with SLE have a significantly increased risk of developing herpes zoster infection compared with the general population; the increased risk was associated particularly with active disease and the use of immunosuppressive therapy.^9,10^

In this study, the aim is to evaluate the safety profile of RZV among SLE patients, including patients on immunosuppressive therapy. Overall, no worsening of lupus disease activity was observed following vaccination. The mean baseline SLEDAI score is (3.43±2.27). Following administration of the first dose of RZV, the mean SLEDI score is (3.48±3.22), while after the second dose, it is (2.97±2.78), which are comparable to the baseline SLEDAI score. The majority of SLE patients (86.5% to 86.6%) show no change in SLEDAI score compared with baseline. Only a few patients (12.2 – 14.8%) experienced a transient increase in SLEDAI score. These results are in line with a recent retrospective study done in the USA, which showed that the RZV administration was not associated with an increased risk of lupus flare-ups or disease exacerbation within 90 days after RZV administration.^8^

Nineteen patients of this study had a documented history of herpes zoster infection prior to receiving RZV. As there is no recommended waiting period between HZ infection and RZV administration, RZV shouldn’t be given in acute HZ infection.^9,10^ From our results, the mean interval between the herpes zoster infection and subsequent RZV administration was 3.5 years, confirming a substantial period of recovery and clinical stability before vaccination. A prospective study showed that the optimal timing for zoster vaccination is one year or more after HZ infection, as natural infection provides only a transient immune response.^13^

In our study, we found that side effects after vaccination were overall mild; the most encountered side effects were local pain and fatigue, while injection site reactions such as swelling and erythema were the least reported. Comparing side effects reported by SLE patients in this study, who are immunocompromised, did not differ from side effects reported by individuals who are immunocompetent in a study conducted in New York.^14^ Another meta-analysis compared the RZV adverse effects between immunocompromised patients and the immunocompetent group, and they found no significant statistical change.^6^

Only two of the patients (1.2%) developed HZ infection post vaccination, suggesting a very low incidence of developing herpes infection after vaccination, which is consistent with a large cohort study that showed a high vaccine effectiveness with an estimate of 78% vaccine efficacy against HZ infection with 2 doses of RZV.^15^

Nearly all patients were receiving at least one immunosuppressive drug at the time of RVZ vaccination, including corticosteroids, with only a minor fraction (6 patients) on higher doses (≥10 mg/day). Our findings suggest that the use of immunosuppressive or **immunomodulatory** biological therapy at the time of vaccination was not associated with increased SLE disease activity or side effects. Because RZV is a non-live vaccine, the guideline supports its use in patients receiving immunosuppressive medications, including biological therapy.^16^ These findings are in line with a study done in Italy, which showed that the RZV vaccine is safe and well tolerated by immunocompromised Rheumatoid Arthritis (RA) patients who are receiving biologic Disease Modifying Anti-Rheumatic Drugs (bDMARDs) therapy.^17^

Vaccinated SLE patients demonstrated stable SLEDAI score across three time points: baseline (3.43 ± 2.27), after the first vaccine dose (3.48 ± 3.22), and after the second dose (2.97 ± 2.78). The difference was not statistically significant (p = 0.133). In contrast, the control group showed a statistically significant reduction in SLEDAI scores over the same period (p = 0.035), particularly between baseline and the second-dose time point. This reduction likely reflects natural disease variability or treatment-related effects rather than vaccine-related changes. A recent retrospective cohort study assessed disease flares among SLE patients in vaccinated and unvaccinated groups and found that vaccinated individuals had no increased risk of severe SLE flares compared with unvaccinated individuals, ^8^ indicating that the administration of RZV did not result in a significant increase in disease activity or flare-ups. Some limitations were encountered in this study; one limitation was that SLE patients with high SLEDAI scores were not included. Another limitation was that not all patients received the full dose of the vaccine at the time of data collection. So, further studies, including a larger number of patients who have completed full vaccination and possibly more active disease, should be carried out.

## CONCLUSION

Our study shows that vaccination with RZV in patients with SLE is safe and well-tolerated. The use of immunosuppressive or biological therapy at the time of vaccination didn’t show an increase in SLEDAI score post vaccination. The reported side effects were mild and comparable to those of the general population.

## Data Availability

All data and materials are available upon request.
